# A Comparison between the Efficacy of Iranian and Syrian Hydrogel Dressings on Wound Healing in Rats

**Published:** 2011-05-01

**Authors:** M H Dashti-Rahmatabadi, M T Noorbala

**Affiliations:** 1 Department of Physiology, Shahid Sadoughi University of Medical Sciencse, Yazd, Iran; 2 Department of Dermatology, Shahid Sadoughi University of Medical Sciencse, Yazd, Iran

**Keywords:** Hydrogel, Dressing, Wound healing, Iran, Syria, Rat

## Abstract

**Background:**

Following the production of Syrian gel, the scientists from Atomic Energy Commission of Syria, researchers in Yazd Atomic Energy Commission Radiation Processing Center in Iran, have manufactured a hydrogel wound dressing. This study was conducted to evaluate and compare the efficacy of these two hydrogels.

**Methods:**

In this study, 32 male Wistar rats underwent a full thickness circular skin wound on the dorsum under light anesthesia. Animals were divided into two groups. Wounds in the 1st group were dressed by the Syrian hydrogel and in the 2nd group by Iranian gel. Wound contraction rate was determined on days 1, 3, 7 and 12 for comparing the wound healing rate in two groups. Laboratory investigation of blood samples, skin tensile strength and histopathology of repaired skins were also evaluated.

**Results:**

Wound contraction ratio on 7th and 12th days in Iranian gel was significantly greater than Syrian gel group. According to histopathological evaluations, wound repair in 63.6% of specimens in Iranian gel group was excellent and in 35.5% good while in Syrian gel group, 54.5% of repaired wounds were graded as excellent and 1 case showed to be in a weak repairing state. A significant difference was noticed in wound repair patterns between the two groups. The differences in skin tensile strength in two groups on days 15 and 30 were not significant.

**Conclusion:**

According to our findings Iranian and Syrian hydrogels did not show any adverse effects on wound healing in rats and could be easily removed from the wound area without any trauma. However Iranian hydrogel dressings were more effective in wound repair regarding wound contraction rate and histopathological evaluation of the skin specimens in the region of healed wounds.

## Introduction

Following the production of hydrogel wound dressings in USA and European countries,[1][2] investigators in many developing countries, such as Indonesia, China, Italy, Japan, Brazil, Malaysia, Philippines, Bangladesh, Syria and Iran, have focused on these products.[1][3] Ajji et al. from Atomic Energy Commission of Syria, introduced their product, in 2004[4] and is now present in the market by the trade name of Syr gel. Syr gel is composed of Polyvinylpyrrolidone (PVP), poly ethylene glycol (PEG) and agar and the gamma ray irradiation technique is used for its polymerization, cross linking and sterilization processes. It has been mentioned that this gel is an excellent barrier against microbes and at radiation dose of 25 kGy is mechanically strength enough for use as wound dressing.[4] Following the production of Syr gel, researchers in Yazd Atomic Energy Commission Radiation Processing Center, to produce a new hydrogel wound dressing with the same composition as the Syr gel but with the substitution of electron beam irradiation technique instead of gammaray irradiation[5] Cyto-toxicity of this product was studied and its safety was approved under ISO 10993 standard in Iran Polymer and Petrochemical Institute.[4]Previously we evaluated the safety and wound healing efficacy of Iranian hydrogel in rats and concluded that the its effect on wound healing was identical to moist gauzes but did not have any adverse effects and was easily removed from the wound surface.[5] Since at the present time, Syr gel is widely used in Iran as a biodegradable wound dressing, in this study we conducted to compare the efficacy of these two hydrogels on the animal model of injured wound.

## Materials and Methods

Thirty two male Wistar rats weighting 200–250 g were provided by the Shaheed Sadughi Medical University Animal House. For experimental procedures we got the permission of the Animal Ethics Committee of Shahid Sadughi Medical University (Yazd, Iran). Experimental procedures were exactly similar to those described in our previous work.[5] Briefly, after shaving and sterilizing the dorsal area, a circular full thickness skin wound was created and disinfected.For wound dressing, animals were randomly and equally divided into 2 groups and their wounds were covered with Syrian or Iranian hydrogel sheets and dressings were changed daily in the morning. The rectal temperature and wound area were measured in the 1st, 3rd, 7th and 12th days of experimental procedure.

Wound contraction ratio (WCR) was calculated according to Balakrishnan et al.[6] On days 15th and 30th, five rats in each group were randomly selected and deeply anesthetized by excess diethyl ether and after taking a blood sample, the animals were sacrificed under diethyl ether over dosage. At the cured wound area, a rectangular piece of skin (1 cm×3 cm) including the entire wound area with adjacent normal tissue was excised for tensile strength measurements and histopathological examinations.

In histopathological evaluation, by considering the integrity of the epithelial surface (partial or complete) and the presence of inflammation (slight, mild, severe), fibrosis (presence or absence), and granulation tissue (presence or absence), each specimen was rated as “excellent” “good” or “weak” repair.[7][8] Blood samples, collected from heart, were assessed by YAZD central lab for routine laboratory tests (CBC, diff, Hb, Hct, urea, cr, SGot, SGpt and Alp). The data were analyzed using a statistical software package (SPSS 11.0 for Windows, Chicago, IL, USA). To compare the histopathological findings, the Chi- Square test was performed. The Student t-test was used for the statistical analysis of wound contraction ratio, body temperature and wound tensile strength. Two-tailed probabilities ≤ 0.05 were considered as the significant level.

## Results

During the experimental evaluation there was no sign of wound infection and no significant changes in mean body temperature among the Syrian and Iranian gel groups (36.8±0.5 vs. 36.9±0.45, p=0.788). Mean percentage of wound contraction on 3rd, 7th and 12th days in Syrian and Iranian gel groups were 18.8±11.4 vs. 22.4±12.8, 54.4±24.6 vs. 70.7±14.6 and 88.3±12.6 vs. 95.5±9.7, respectively and there was a significant difference among two groups in 7th and 12th days (p=0.015 and p=0.036 respectively).

**Fig. 1:**
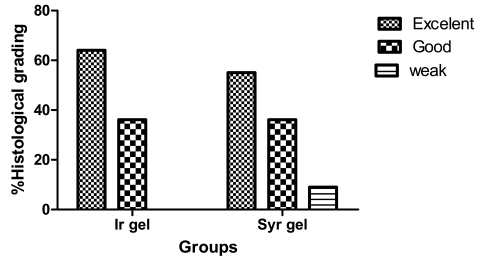
Wound repairing grades (WRG) in histological examination of healed wound specimens in Syrian hydrogel (Syr gel) and Iranian hydrogel (Ir gel) groups (n=5). Statistical analysis, using Fisher's Exact test showed a significant difference between 2 groups (p=0.008)

In the evaluation of tensile strength, the mean maximum possible tensile length of healed wounds (breakdown length) on day 15 was 26.2±4.1 mm in the Syrian gel group and 27.8±9.8 mm in the Iranian gel group and the difference was not significant (p=0.421). On day 30, also there was no significant deference in the index of tensile strength among Syrian gel and Iranian gel groups. Healed wound specimens were histologically examined and graded into 3 different categories, excellent, good or weak repair. Wound repair in 54.5% of specimens in Syrian gel group and 63.6% in Iranian gel group were excellent and only 1 case (9.1%) in Syrian group showed to be in a weak repairing state. Statistical analysis using Chi-Square test showed a significant difference in wound repairing pattern between the two groups (p=0.008) (Figure 1). Blood sample assessment showed that all blood parameters were within the normal limits in both groups.

## Discussion

According to this study, wound contraction ratio in Iranian gel group on 7th and 12th days were significantly greater than that of Syrian gel group (p=0.015 and p=0.036 respectively). The histopathological evaluation also revealed a significant difference in wound repairing pattern between two groups (p=0.008). Although, some characteristics of hydrogel dressings are evaluated (mainly by the manufacturers),[9] there is a few published data concerning the evaluation of efficacy and safety of these sheets on wound healing. Mulder et al. evaluated the safety, efficacy, and functional attributes of clearsite hydrogel dressing (New Dimensions in Medicine, Dayton, Ohio, USA) as compared with standard wet-tomoist gauze dressing and DuoDERM hydrocolloid dressing (ConvaTec/Bristol Myers-Squibb, Princeton, NJ, USA) and concluded that there was no statistically significant difference in wound healing rate among the three treatments.[10]Negishi et al. assessed the clinical effectiveness and safety of hydrogel wound dressing for the treatment of venous stasis ulcers and concluded that the treatment with hydrogel dressing was safe and effective for venous ulcers of the lower limbs.[11] Jones and Vaughanthe in a review article discussed some differences between 7 different types of hydrogel products and their implications for clinical practice. This review supports the effectiveness of these products in accelerating natural debridement, moistening and hydrating necrotic tissues and loosening and absorbing sloughs and exudates in a variety of skin wounds.[12]

The manufacturers of new Iranian hydrogel indicated that its gel fraction is generally lower than the Syrian gel but their maximum swelling rate is identical.[4] However swelling kinetics of Iranian hydrogel vs. the time was greater than Syrian gel but its dehydration rate was slower.[4]According to our findings, although the tensile strength of the healed wound on day 15 and 30, in Iranian gel was the same as Syrian gel, the mean percentage of wound contraction on 7th and 12th days of treatment in Iranian gel group was significantly greater than the Syrian gel and in the case of histopathological evaluation, wound repairing pattern in Iranian gel was better than the Syrian gel. These differences may be due to the different gelation rate and water content of these two types of hydrogel dressings which can affect the transmission of oxygen and water vapor to the wound surface.[13] It has been shown that moistening the wound surfaces facilitates the formation of granulation tissue.[10]Also, it has been mentioned that wound contraction rate is largely influenced by fibroblast function and collagen formation in the wound area,[9] which needs to be further investigated. The present study also supports the safety of two hydrogels when evaluated visually and also did not show any adverse effect on vital organs and were not pyrogenic. Since the major difference in manufacturing processes of these two gel types is in irradiation technique, we propose further investigations regarding the effect of various irradiation sources, used in cross linking the gels, on the efficacy of hydrogel wound dressings.
